# Clinical features of renal damage associated with Epstein-Barr virus infection in children

**DOI:** 10.3389/fped.2023.1123941

**Published:** 2023-03-23

**Authors:** Xiaoqing Yang, Baohua Lin, Tong Shen

**Affiliations:** ^1^ Pediatrics Department, Women and Children’s Hospital, School of Medicine, Xiamen University, Xiamen, China; ^2^ Xiamen Maternal and Child Health Care Hospital, Xiamen, China

**Keywords:** Epstein-Barr virus, infectious mononucleosis, renal damage, children, hematuria

## Abstract

**Objective:**

To understand the renal damage and clinical features of pediatric patients with acute Epstein-Barr virus (EBV) infection

**Methods:**

In this retrospective observational study, 548 pediatric patients who were admitted to and treated at the Xiamen Women and Children Health Center between January 2017 and December 2021 and who met the criteria of acute EBV infection were selected as participants. The sociodemographic and clinical data of these patients were collected for statistical analysis. The study population was divided into a renal damage group (41 patients) and a non-renal damage group (507 patients), and the characteristics of the two groups were compared.

**Results:**

(1) Of the 548 enrolled patients, 340 and 208 were boys and girls, respectively. Among them, 41 patients showed renal damage (renal damage group), including 26 boys and 15 girls, and the incidence rate of renal damage was 7.48%. (2) The major renal involvements in the 41 pediatric patients with acute EBV infection in the renal damage group manifested as hematuria (56.1%), proteinuria (37.71%), hematuria + proteinuria (12.9%), edema (51.22%), hypertension (17.07%), oliguria (4.88%), and acute renal failure (2.44%). (3) The pediatric patients in the renal damage group had statistically significantly longer fever durations, higher blood EBV-DNA loads, and lower blood CD4^+^/CD8^+^ T lymphocyte ratios than those in the non-renal damage group.

**Conclusion:**

In pediatric patients, the incidence rate of acute EBV-induced renal damage is not low. The clinical manifestations are mostly hematuria or proteinuria, with an overall good prognosis, but occasionally severe renal damage such as acute renal failure. The possibility of secondary renal damage is high when pediatric patients with acute EBV have prolonged fever, high blood EBV-DNA loads, and decreased blood CD4^+^/CD8^+^ ratios.

## Introduction

The Epstein-Barr virus (EBV) belongs to the human herpes virus type IV. It was discovered and named in 1964 by two researchers, Epstein and Barr, studying in-vitro lymphoma cell cultures of children with malignant lymphoma. EBV infection is common in the healthy population. Infectious mononucleosis and asymptomatic infection are the most common forms of primary EBV infections in children. Previous studies have reported that EBV infection can involve various organs such as those of the respiratory, digestive, hematological, or nervous system. Among them, renal damage is often overlooked. EBV infection can manifest with different symptoms such as hematuria, proteinuria, nephrotic syndrome, and even renal failure. Owing to the varying severity and lack of specificity of the symptoms, EBV infections are poorly recognized and underreported, especially in children. Here, we review and analyze the data of 548 pediatric patients with acute EBV infections in our hospital within the 5 years from January 2017 to December 2021. Varying degrees of renal involvement were found in 41 of these patients. We intend to summarize the clinical features and prognosis of EBV infection-associated renal damage in children to improve the understanding of this complication.

## Data and methods

### Study population

The clinical data of the study participants, comprising 548 pediatric patients who met the criteria of acute EBV infection and who were admitted to and treated at the Xiamen Maternal and Child Health Care Hospital between January 2017 and December 2021, were collected. This study was approved by the Medical Ethics Committee of Xiamen Maternal and Child Health Care Hospital [approval number: FY-20161008]. All parents or legal guardians of the enrolled children signed the informed consent form.

### Diagnostic criteria

Individuals were included in the study based on the diagnostic criteria for acute EBV infection as described in the Expert Consensus on the Principles of Diagnosis and Treatment of EBV Infection-Related Diseases in Children (1). Thus, we enrolled pediatric patients with any three of the clinical manifestations of fever, pharyngitis, large cervical lymph nodes, hepatomegaly, splenomegaly, rash, and eyelid edema who were positive for anti-EBV capsid antigen (CA)-IgM and anti-EBV CA-IgG but negative for EBV nuclear antigen (NA)-IgG, or negative for EBV CA-IgM but positive for EBV CA-IgG with low-affinity antibodies. Renal involvement was defined as hematuria (>3 red blood cells per high-power field in the microscopic examination of urine sediment), proteinuria (positive in the qualitative urine protein test or a urine protein quantification of more than 150 mg per day), and elevated blood creatinine levels (CREA >100umol/L).

Exclusion criteria were: (1) pediatric patients co-infected with other pathogens such as other viruses, mycoplasma, bacteria, and fungi; (2) pediatric patients with underlying renal diseases; and (3) pediatric patients with severe underlying diseases of important organ systems, e.g., respiratory, digestive, neurological, or immune disorders.

### Research methods

The data of the pediatric patients such as sociodemographic information (age and sex), hospital stay, clinical manifestations, results of auxiliary examinations, and treatment plan were recorded by reviewing their medical records. The pediatric patients were divided into renal damage and non-renal damage groups according to the presence or absence of renal damage. Differences in relevant data were compared between the two groups of pediatric patients. The clinical and laboratory characteristics of the renal damage in acute EBV infection were also analyzed.

### Statistical methods

The statistical analysis was performed using SPSS 22.0 software. Normally distributed data were expressed as (*x* ± *s*) whereas non-normally distributed data were expressed as median (minimum, maximum). The *t*-test was used for the comparison of means, whereas the rank sum test and *χ*^2^ test were used for the comparison of medians and rates (composition ratio), respectively. Differences were considered statistically significant if *P *< 0.05.

## Results

### General information

A total of 548 pediatric patients with acute EBV infections who had been admitted to and treated at the Xiamen Women and Children Health Center in the 5-year period from January 2017 to December 2021 met the inclusion criteria. This study population included 340 boys and 208 girls, with a median age of 5 years and 7 months (ranging from 1 year and 1 month to 12 years and 3 months). Among them, 41 patients developed renal damage (renal damage group), including 26 boys and 15 girls, with a median age of 4 years and 11 months (ranging from 1 year and 8 months to 10 years and 9 months), including 11 patients with an age <3 years, 18 patients in the age range of 3–6 years, and 12 patients with an age >6 years. The incidence of renal damage was 7.48%.

### Clinical characteristics, auxiliary examinations, and disease progression of pediatric patients in the renal damage group

The major renal involvements in the 41 patients of the renal damage group manifested as hematuria, proteinuria, edema, and hypertension. All signs and symptoms of renal damage occurred within 1 week of the EBV infection. The details are as follows (see Table 1): 23 cases of simple hematuria (including 19 cases of microscopic hematuria and 4 cases of macroscopic hematuria); 13 cases of proteinuria (including 2 cases of massive proteinuria); 5 cases of hematuria + proteinuria; 21 cases of edema; 2 cases of lower back pain; 7 cases of hypertension; and 2 cases of oliguria and elevated blood creatinine levels. Of the 41 pediatric patients, 33 underwent renal color Doppler ultrasound examination, and the results showed that 7 patients had enlarged kidney volume, and 3 patients had varying degrees of peritoneal effusion. One pediatric patient underwent a renal biopsy, and the pathological results showed minimal change disease (MCD).

**Table 1:** Manifestations of renal damage in 41 pediatric patients with acute EBV infection.

Item	*n*	%
Hematuria	23	56.1
Proteinuria	13	31.71
Hematuria + proteinuria	5	12.19
Hypertension	7	17.07
Edema	21	51.22
Oliguria	2	4.88
Elevated blood creatinine level	2	4.88
Abnormal finding in renal color Doppler ultrasound	7	17.07
Acute renal failure	1	2.44

EBV, Epstein-Barr virus.

The 41 patients of the renal damage group were treated symptomatically, e.g., with rehydration, fever reduction, and liver protection (Glutathione). Acyclovir or ganciclovir antiviral therapy was administered to 29 of these patients. Of the 41 patients, 38 were discharged from the hospital completely cured. Two pediatric patients who had developed nephrotic syndrome were treated with adequate glucocorticosteroids and became negative for urine protein within 1–2 weeks. Another patient who had EBV-related phagocytic syndrome combined with acute renal failure recovered after being given acyclovir antiviral therapy combined with an immunochemotherapy regimen, as well as comprehensive treatment such as water restriction and correction of acidosis and hyperkalemia. All patients were followed up for 6–12 months after discharge, and no abnormalities were observed. For the patient with EBV infection combined with phagocytic syndrome and acute renal failure, the results of the long-term follow-up showed that this patient was treated at the outpatient clinic and hospitalized due to acute severe infection 3 years after discharge. The infection was poorly controlled, leading to multisystem organ failure and eventually death. The primary cause of death was acute renal failure.

### Comparison of clinical characteristics between the renal damage and non-renal damage groups

Of the 548 enrolled patients, 507 were in the non-renal damage group, including 314 boys and 193 girls. The median age in this group was 5 years and 6 months (ranging from 1 year and 1 month to 12 years and 3 months), including 187 patients with an age <3 years, 229 patients in the age range of 3–6 years, and 91 patients with an age >6 years. The incidence rates of pharyngitis and lymph node enlargement were 92.9% and 84.02%, respectively. In this non-renal damage group, the proportion of heteromorphic lymphocytes was 14.23 ± 4.79%. For the 41 cases in the renal damage group, the incidence rates of pharyngitis and lymph node enlargement were 95.12% and 87.81%, respectively. The proportion of heteromorphic lymphocytes was 13.86 ± 4.64% in the renal damage group. These clinical data were not significantly different between the two study groups (all *P *> 0.05). The duration of fever was 5.02 ± 1.93 days in the non-renal damage group but significantly longer with 7.29 ± 2.59 days in the renal damage group (*P *< 0.05). Moreover, EBV-DNA copies/mL differed significantly between the two groups of pediatric patients (*P *< 0.05; see Table 2).

**Table 2:** Comparison of clinical data between the non-renal damage and renal damage groups.

Item	Non-renal damage group (*n* = 507)	Renal damage group (*n* = 41)	*χ*^2^/*t*	*P*
Sex (*n*)
Male	314	26	0.035	0.851
Female	193	15		
Age (*n*)
<3 years	187	11	3.661	0.16
3–6 years	229	18		
>6 years	91	12		
Fever (days)	5.02 ± 1.93	7.29 ± 2.59	7.024	0.016
Pharyngitis, *n* (%)	471 (92.9%)	39 (95.12%)	0.29	0.59
Lymph node enlargement, *n* (%)	426 (84.02%)	37 (87.81%)	0.41	0.522
Percentage of heteromorphic lymphocytes (%)	14.23 ± 4.79	13.86 ± 4.64	0.465	0.642
EBV-DNA copies/ml (*n*)
10^3^–10^5^	398	24	8.539	0.003
≥10^6^	109	17		

EBV, Epstein-Barr virus.

A total of 119 pediatric patients completed the immunoglobulin and peripheral blood lymphocyte subset (CD series) tests, including 28 of the renal damage group and 91 of the non-renal damage group. The differences in IgM, IgA, and IgG levels between the two groups were not statistically significant (all *P *> 0.05). By contrast, the difference in CD4^+^/CD8^+^ ratios between the two groups was statistically significant (*P *< 0.05; see Table 3).

**Table 3:** Comparison of immune function between the non-renal damage group and the renal damage group.

	*n*	IgA (g/L)	IgG (g/L)	IgM (g/L)	CD4/CD8
		x ± s	x ± s	x ± s	Median (p25, p75)
Non-renal damage group	91	3.02 ± 1.22	9.39 ± 3.21	2.06 ± 0.81	1.21 (0.65, 2.17)
Renal damage group	28	2.59 ± 1.10	9.87 ± 2.74	1.74 ± 0.83	0.84 (0.42, 1.10)
*t/z*		1.264	−0.54	1.414	−2.502
*P*		0.211	0.591	0.162	0.012

## Discussion

EBV infection is common in children and can involve multiple organ systems of the body, including the kidneys. Although all levels of healthcare professionals pay increasing attention to the issue of EBV infection-induced renal damage, global studies reporting definitive incidence rates of EBV infection-associated renal damage are still lacking. Currently, the reported incidence rates of EBV infection-associated kidney damage vary widely, with the literature showing incidence rates ranging from 1.7% to 27.5% (2–6). Overall, the reported incidence rates in recent years seem to be higher than those in the past. The incidence rate of EBV infection-associated kidney damage in this study was 7.48%, which was well within the previously reported range. However, regardless of the magnitude of the incidence rate, the increasing number of reported cases of EBV infection-associated kidney damage suggests that we should pay sufficient attention to this problem.

In this study, the manifestations of EBV infection-induced renal damage lacked specificity and mainly presented as varying degrees of hematuria or proteinuria, as well as edema and hypertension. The predominant manifestation of renal damage in our study was microscopic hematuria, with hematuria alone accounting for 56.1% of all clinical manifestations and up to 68.29% when including individuals with hematuria combined with proteinuria. Thus, hematuria may be the predominant manifestation of EBV infection-associated renal damage, and other reports from around the world came to similar conclusions (2, 5, 6). However, a few studies have stated that proteinuria is the predominant renal manifestation of this disease (3, 4). The definitive incidence rates of manifestations such as hematuria or proteinuria still need to be further studied with expanded sample sizes. In this study, 38 pediatric patients (92.68%) had renal manifestations that gradually resolved in about 1 week with an overall good prognosis, but two patients developed proteinuria resulting from renal disease. Another pediatric patient developed EBV-associated phagocytic syndrome combined with acute renal failure. Although this patient improved with aggressive treatment, it suggests that EBV infection can cause serious renal disorders such as renal failure. In addition, long-term follow-up showed that this pediatric patient died 3 years later from acute renal failure induced by a severe infection. The relationship between the re-emerging fatal renal failure and the EBV infection from several years ago evokes associations with previously reported cases. A researcher from a hospital in Taipei, China reported eight cases of acute renal failure in EBV-infected pediatric patients within 6 years, and two of these patients eventually died (7). This suggests that EBV infection-induced severe kidney damage is not uncommon and can seriously affect the prognosis if not treated aggressively. The proportion of patients with EBV infection-induced renal damage who complete a renal biopsy is low. At present, it is believed that it can present different pathological types, such as interstitial nephritis, membranoproliferative glomerulonephritis, and minimal change disease (MCD). Moretti et al. (8) reported 27 cases of EBV infection-induced renal damage that completed a renal biopsy, and about 50% (13/27) of the cases showed typical changes indicative of interstitial nephritis. In the present study, only one pediatric patient underwent a renal biopsy, and the results showed microscopic lesions. This suggests that aside from damaging predominantly the renal interstitium, EBV may also affect the renal parenchyma and pedicles.

The exact mechanism of EBV-induced renal damage is not fully understood, but it is thought to originate from two pathological mechanisms. First, EBV itself directly attacks the kidneys. Previous studies have shown that the virus can enter renal cells (glomeruli, tubules, and interstitial cells) and causes functional and structural damage to these cells through viral replication. Becker et al. (9) reported that EBV can directly invade renal parenchymal cells with the help of its cellular receptor, the CD21 antigen. Okada (10) detected actively replicating EBV-DNA in renal tissues as direct evidence. Second, renal damage is thought to derive from EBV-mediated immunological injury. Physicians observed considerable renal interstitial edema and numerous lymphocyte and plasma cell infiltrations in the renal biopsy tissues of patients with EBV-induced renal damage, which are the typical histological manifestations of immune complex-mediated renal damage. Immunohistochemical stainings confirmed both the predominance of CD20^+^ B lymphocytes and EBV antigen positivity in the renal tissues of patients with organ transplantation and EBV infection (11, 12).

The present study compared also the clinical data of two groups of acute EBV-infected pediatric patients, those with and those without renal damage. The results showed no statistical differences between the two study groups in terms of age at the onset of disease, sex, incidence rates of pharyngitis and lymph node enlargement, and proportion of heteromorphic lymphocytes, corroborating globally reported findings. However, the duration of fever differed between the two groups of pediatric patients; it was significantly longer in pediatric patients with renal damage than in those without. This study also found that the EBV-DNA load was higher in pediatric patients of the renal damage group than in those of the non-renal damage group. This indicates that pediatric patients with high EBV-DNA load are more likely to be affected by renal damage. The long duration of fever and the high EBV-DNA load suggest a strong inflammatory response in EBV-infected patients. We believe that viremia caused by the massive replication of the virus and the infiltration of numerous abnormal lymphocytes into the kidneys of the patients during this process are the potential causes of direct cellular damage in the affected kidneys.

The present study also showed that the blood CD4^+^/CD8^+^ T cell ratios of the pediatric patients in the renal damage group were lower than those in the non-renal damage group, suggesting a link between the development of renal damage and the suppression of cellular immune function following acute EBV infection. EBV infection-induced immunological liver damage has been more intensively studied. It is now believed that EBV attacking B lymphocytes leads to their antigenic alteration, which in turn activates T lymphocytes transforming them into cytotoxic T lymphocytes that destroy EBV-carrying B lymphocytes. Liver cell damage is then induced by the toxic effects of lipid peroxidation and free radicals (13, 14). The immune disorders of T and B lymphocytes mentioned above are likely to contribute to the immune basis of renal damage in EBV-infected pediatric patients. However, due to limitations regarding the incidence rate and degree of severity of renal damage in EBV infection, the current evidence on the association of immune abnormalities in EBV-infected renal tissues with EBV infection is still limited. Further studies with expanded samples are needed in the future.

In conclusion, the incidence rate of acute EBV infection-induced renal damage is not low in pediatric patients. The clinical manifestations are mostly hematuria or proteinuria, with an overall good prognosis but occasionally severe renal damage such as acute renal failure. Secondary renal damage is more likely when pediatric patients with acute EBV infection have prolonged fever, high blood EBV-DNA loads, and decreased blood CD4^+^/CD8^+^ ratios.

## Data Availability

The original contributions presented in the study are included in the article/Supplementary Material, further inquiries can be directed to the corresponding author.
